# Incidence and Risk Factors for Early Acute Kidney Injury in Nonsurgical Patients: A Cohort Study

**DOI:** 10.1155/2017/5241482

**Published:** 2017-04-11

**Authors:** Javier Enrique Cely, Elkin José Mendoza, Carlos Roberto Olivares, Oscar Julián Sepúlveda, Juan Sebastián Acosta, Rafael Andrés Barón, Juan José Diaztagle

**Affiliations:** ^1^Department of Internal Medicine, Fundación Universitaria de Ciencias de la Salud, San Jose Hospital, School of Medicine, Bogotá, Colombia; ^2^Department of Nephrology, Dialysis and Transplantation, Fundación Universitaria de Ciencias de la Salud, San Jose Hospital, School of Medicine, Bogotá, Colombia; ^3^Department of Physiological Sciences, School of Medicine, Universidad Nacional de Colombia, Bogota branch, Bogotá, Colombia

## Abstract

*Introduction*. Detecting acute kidney injury (AKI) in the first days of hospitalization could prevent potentially fatal complications. However, epidemiological data are scarce, especially on nonsurgical patients.* Objectives*. To determine the incidence and risk factors associated with AKI within five days of hospitalization (EAKI).* Methods*. Prospective cohort of patients hospitalized in the Internal Medicine Department.* Results*. A total of 16% of 400 patients developed EAKI. The associated risk factors were prehospital treatment with nephrotoxic drugs (2.21 OR; 95% CI 1.12–4.36, *p* = 0.022), chronic kidney disease (CKD) in stages 3 to 5 (3.56 OR; 95% CI 1.55–8.18, *p* < 0.003), and venous thromboembolism (VTE) at admission (5.05 OR; 95% CI 1.59–16.0, *p* < 0.006). The median length of hospital stay was higher among patients who developed EAKI (8 [IQR 5–14] versus 6 [IQR 4–10], *p* = 0.008) and was associated with an increased requirement for dialysis (4.87 OR 95% CI 2.54 to 8.97, *p* < 0.001) and in-hospital death (3.45 OR; 95% CI 2.18 to 5.48, *p* < 0.001).* Conclusions*. The incidence of EAKI in nonsurgical patients is similar to the worldwide incidence of AKI. The risk factors included CKD from stage 3 onwards, prehospital treatment with nephrotoxic drugs, and VTE at admission. EAKI is associated with prolonged hospital stay, increased mortality rate, and dialysis requirement.

## 1. Introduction

Acute kidney injury (AKI) has a high impact on healthcare systems because of its high morbidity and mortality rates, length of hospital stay, and treatment costs [1–3]. Thus, prevention and early diagnosis are essential to provide measures to avoid the onset of dialysis as much as possible. Although molecular markers of early kidney damage would be ideal [4], they are, unfortunately, unavailable for routine clinical use. Therefore, variations in serum creatinine according to the Acute Kidney Injury Network (AKIN) and Kidney Disease Improve Global Outcome (KDIGO) criteria remain a valid tool for diagnosis [5, 6].

The mission of healthcare institutions is to know local epidemiology and to generate prevention strategies based on the knowledge of risk factors, which should be identified early upon hospital admission, towards eradicating in-hospital preventable deaths from AKI [7]. Such factors have already been reported in previous publications and are best known in the septic population and within intensive (ICU) and postoperative (PCU) care units, among others [8–10]. Specifically, conditions such as diabetes, proteinuria, and reduced renal function on admission have been reportedly linked to the development of AKI in patients with severe sepsis [11]. However, we do not know whether these criteria apply to other scenarios, including nonsurgical patients and during early hospitalization.

To date, there have been no identifiable studies that evaluate variables related to the development of EAKI in this subset of nonsurgical patients who are managed by an Internal Medicine team. The importance of these studies lies in providing a useful tool for clinicians to prevent the progression of the disease and to help avoid morbid treatments such as dialysis. Therefore, the present study aims to assess the incidence and risk factors associated with the development of AKI in nonsurgical patients, including a population with CKD and at early stages of hospitalization.

## 2. Materials and Methods

### 2.1. Study Design and Patients

A prospective cohort study was performed at the San Jose Hospital in Bogota Colombia, a level-four university hospital that provides healthcare to more than 2,500 patients per year in the Internal Medicine Department and has a Nephrology and Dialysis Department and a transplantation unit. We included adult patients admitted for emergency care and hospitalized in the Internal Medicine Department for more than 48 hours from September 2015 to April 2016. Patients on chronic dialysis or meeting the criteria for urgent dialysis on admission, pregnant, of history of kidney transplantation, of community-acquired acute kidney injury (CA-AKI), or transferred to the ICU within 48 hours were excluded from the study. Creatinine levels were measured on admission, at 48 hours and on day 5 of hospital stay, to establish the presence of AKI, based on the following operational definitions.

*EAKI*. Patient admitted with normal creatinine levels (the creatinine reference values were used according to the local clinical laboratory men ≤ 1.3 mg/dL and women ≤ 1.1 mg/dL) and with an increase in creatinine equal to or greater than 0.3 mg/dL when comparing creatinine on admission with the control at 48 hours or on day five (based on the diagnostic recommendations of the KDIGO guidelines for AKI, the criterion based on changes in urinary output was not considered) [6].

*CA-AKI*. Patients with increased creatinine on admission and some of the following conditions:

- An increase ≥0.3 mg/dL creatinine on admission compared with a prehospital record of creatinine six months before admission
- If no previous record exists, the evaluation group was responsible for the clinical and paraclinical assessment (i.e., renal diagnostic imaging, abnormal bone mineral metabolism, or other findings suggestive of CKD) to define CA-AKI or CKD without AKI
- Creatinine levels at the end of hospitalization lower than the creatinine levels on admission with a difference ≥0.3 mg/dL

### 2.2. Source and Monitoring Methods

Data were collected from electronic medical records and were corroborated by direct patient examination. A project coordinator was responsible for conducting the daily patient census and assessing the eligibility criteria and monitoring. In case of doubt, a medical research group formed by three nephrologists (evaluation group) performed the final patient classification.

### 2.3. Variables

Clinically relevant variables included history of AHT, DM, heart failure, cirrhosis, coronary heart disease, rheumatologic disease, nephrotic syndrome, and CKD. According to the National Kidney Foundation Kidney Disease Outcomes Quality Initiative (NKF KDOQI) guideline, CKD was defined as GFR ≥ 60 mL/min/1.73 m^2^ with structural or functional kidney abnormality (abnormal composition of urine and/or abnormal imaging studies) for ≥3 months or GFR < 60 mL/min/1.73 m^2^ with or without kidney damage for ≥3 months [12]. Similarly, CKD was staged from 1 to 5 according to the NFK-KDOQI guideline with the GFR estimated by the CKD-EPI equation using the creatinine on admission [13]. Stages 1 and 2 were analyzed as a single variable and stages 3, 4, and 5 as another variable for two reasons: first, the number of patients with CKD in stages 1, 4, and 5 was poor in the cohort and, secondly, the risk of AKI in patients with reduced GFR from 60 mL/min/1.73 m^2^ (stages 3, 4, and 5) is better known in previous publications; however, it is uncertain for stages 1 and 2 (GFR ≥ 60 mL/min/1.73 m^2^ with structural or functional kidney abnormality) [14].

Other variables of interest assessed included age, sepsis, hydration status on admission based on the attending physician's criteria, main diagnosis on admission, prehospital and in-hospital treatment with nephrotoxic drugs shown in Table 1 (the operational definitions for nephrotoxic drugs are shown in Table 1 of Supplementary Material available online at https://doi.org/10.1155/2017/5241482), ICU admission after 48 hours of hospital stay, dialysis requirement, length of hospital stay (including days of ICU stay if admitted 48 hours after hospital admission), and in-hospital death.

### 2.4. Sample Size

Sample size calculation was performed using a logistic regression model based on a prevalence of hospital-acquired AKI of approximately 17.2% [15, 16] and expecting to obtain at least 10 events for each of the five covariates considered to be the most important risk factors: CKD at admission, administration of nephrotoxic drugs, age, history of diabetes mellitus (DM), and sepsis [10, 11, 14]. The result is a minimum required sample size of 348 patients.

### 2.5. Statistical Methods

A database was constructed and statistical analysis was performed using STATA 13®. Descriptive statistics were used to report the absolute and relative frequencies of categorical variables, and measures of central tendency and dispersion were used for quantitative variables, considering their distribution based on the Shapiro-Wilk test. The Mann–Whitney *U* test was used for quantitative variables with abnormal distribution. The incidence of EAKI was calculated.

A bivariate analysis was performed to assess the relationship between independent variables (exposure) and EAKI using the Chi-squared (*χ*^2^) test. Subsequently, a multivariate analysis by logistic regression model with odds ratio (OR) calculation was done. All clinically relevant variables and those with *p* values < 0.1 were included in the analysis. A *p* value < 0.05 was considered significant in the multivariate analysis. Variables without relevance to the model were removed using a backward strategy. The goodness of fit was based on Hosmer and Lemeshow criteria.

### 2.6. Ethics

Ethical principles of the Declaration of Helsinki and Colombian regulations issued by the Ministry of Health pursuant to resolution 8430 of 1993 were considered. The protocol was approved by the Research Committee of the School of Medicine of the Foundation University of Health Sciences and the Ethics Committee on Human Research of San Jose Hospital, Bogotá.

## 3. Results

**Figure 1:**
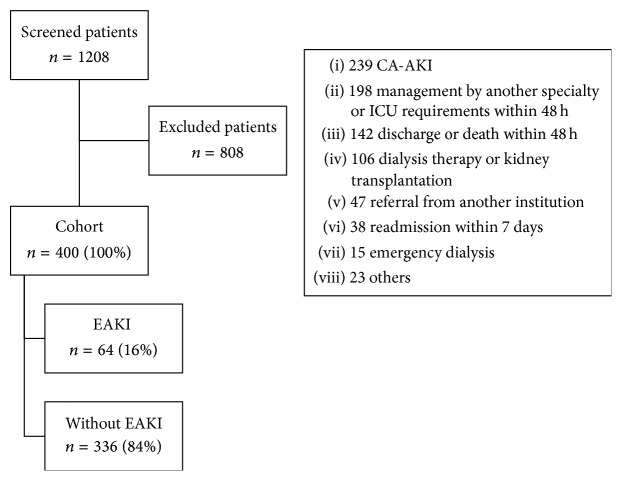
Cohort selection process.

A total of 1,208 patients were evaluated during the collection period, including 400 who met the inclusion criteria (Figure 1). A total of 55% (*n* = 220) were women, the median age was 65 years (IQR 49–77), and the median creatinine levels on admission were 0.9 mg/dL (IQR 0.7–1). The most common diagnosis on admission was bacterial infection and the most common comorbidity was AHT followed by DM (Table 1). Some 16% (*n* = 64) of the population developed EAKI, classified as 84.4% KDIGO 1 (*n* = 54), 12.5% KDIGO 2 (*n* = 8), and 3.1% KDIGO 3 (*n* = 2), depending on the severity of renal injury [6].

The following variables were associated with the development of EAKI in the bivariate analysis. Age (OR 1.02; 95% CI 1.00 to 1.03, *p* = 0.019), CKD on admission stages 3, 4, and 5 (OR 4.00; 95% CI 2.13 to 7.45 *p* < 0.001), history of DM (OR 2.06; 95% CI 1.09 to 3.80), history of AHT (OR 1.82; 95% CI 1.02 to 3.28, *p* = 0.030), and prehospital treatment with nephrotoxic drugs (OR 2.63; 95% CI 1.38 to 5.25, *p* = 0.002) (Table 2). The individual analysis of each nephrotoxic drug revealed that prehospital (OR 2.31; 95% CI 1.22 to 4.30, *p* = 0.004) or in-hospital (OR 2.24; 95% CI 1.24 to 4.00, *p* = 0.003) treatment with furosemide was associated with EAKI.

In the logistic regression EAKI was associated with CKD on admission stages 3, 4, and 5 (OR 3.56; 95% CI 1.55 to 8.18, *p* = 0.003), prehospital treatment with nephrotoxic drugs (OR 2.21; 95% CI 1.12 to 4.36, *p* = 0.022), and venous thromboembolism (OR 5.05; 95% CI 1.59 to 16.0, *p* = 0.006). (Table 3).

Regarding outcomes, the overall mortality rate was 7.5% (*n* = 30); the mortality rate among patients with EAKI was 21.9% (*n* = 14), and the following mortality rates were assessed according to the KDIGO criteria of AKI severity: 18.5% (*n* = 10) for KDIGO 1, 25% (*n* = 2) for KDIGO 2, and 100% (*n* = 2) for KDIGO 3. Increased associations with in-hospital death (OR 5.6; 95% CI 2.36 to 13.0, *p* < 0.001) and dialysis requirement (OR 16.5; 95% CI 1.28 to 867.2, *p* = 0.0012) occurred among patients who developed EAKI. The median length of hospital stay of patients with EAKI was 8 (IQR 5–14), in contrast to 6 (IQR 4–10), among patients who did not develop the condition (*p* = 0.008) (for details on hospital stay by EAKI and death, see Table 2 of the Supplementary Material).

## 4. Discussion

The present cohort study aimed to estimate the incidence of AKI in nonsurgical patients detected during early hospitalization and to identify the associated risk factors. Under routine clinical measures, our patients apparently did not have AKI on admission. The present study showed that 16% of nonsurgical patients developed AKI within five days of hospitalization. Such a finding is difficult to interpret when compared with the international literature. First, the operational definitions of AKI vary by year of publication (especially before or after the RIFLE consensus, AKIN, and KDIGO) [5, 17, 18]. Second, each clinical stage and study population is different (e.g., medical and/or surgical and CA-AKI and ICU patients), and AKI was only assessed during early hospitalization without identifying the incidence throughout the hospital stay.

The worldwide pooled incidence rates of hospital-acquired AKI range from 17.2% to 25.2%, with high heterogeneity among the studies analyzed in the meta-analysis by Susantitaphong et al. [15]. This incidence is similar to that reported in the present cohort study, despite including no surgical patients and only cases of AKI diagnosed within five days of hospitalization. Therefore, patients managed by the Internal Medicine specialty without apparent AKI on admission may be a population vulnerable to the development of AKI during early hospitalization, possibly because AKI patients are older and have high associated morbidity, which underlines the importance of performing early screening and monitoring in the first days of hospitalization.

Conversely, 84% of EAKI cases in the present study correspond to a KDIGO 1 (“mild” AKI) classification, with an 18.5% mortality rate, which highlights the existing link between slight increases in serum creatinine levels and mortality rate, as shown by other authors [19–21]. This issue is controversial, given the recent evidence suggesting the existence of a high rate of false-positive AKI cases only diagnosed based on an absolute increase in creatinine levels ≥0.3 mg/dL, particularly in a population with CKD [22]. Furthermore, these values may be affected by hydration status and/or fluid replacement without implying AKI [23]. This dilemma will most likely be resolved when we have the capacity to routinely use molecular markers of EAKI [4, 24].

The seminal studies by Hou et al. [25] and Shusterman et al. [26] showed that the volume depletion, treatment with aminoglycosides, contrast media, heart failure, and septic shock increased the risk for AKI in a medical-surgical population. However, it should be considered that those studies were conducted at least three decades ago, using different AKI definition criteria from those currently used, only detecting patients with severe renal injury and overlooking those with a lesser degree of renal injury. In contrast, treatment with aminoglycosides is uncommon in the Internal Medicine Department, thus lacking a key role in the present cohort study. Conversely, the failure to identify a relationship between EAKI and the administration of nephrotoxic drugs could be explained because the condition was only assessed within five days of hospitalization and requires later screening to be detected by variations in serum creatinine levels. However, we do not rule out the hypothesis that the attending physicians were sensitized at the beginning of our study, which may have changed their nephrotoxic drug prescribing habits.

However, the main risk factor for developing EAKI on admission is CKD from stage 3 onwards. This association between AKI and CKD is quite complex and has been well described. CKD increases the risk for AKI [14, 27–29]; AKI causes CKD, and both entities share risk factors for its development [30, 31]. The results from our cohort study corroborate such a relationship between the two entities and highlight the importance of screening and preventive measures in patients with decreased GFR on admission.

Another important aspect to discuss is the scarce literature that has assessed AKI risk in patients with CKD stages 1 and 2 (GFR ≥ 60 mL/min/1.73 m^2^ with structural or functional kidney abnormality), being uncertain of the role of these early stages as a risk factor for AKI. Even in risk studies of AKI in CKD, such as that by Hsu et al., GFR ≥ 60 mL/min/1.73 m^2^ is the point of reference of “normality” to be compared with those having GFR < 60 mL/min/1.73 m^2^. No relation was found between stages 1 and 2 of CKD (GFR ≥ 60 mL/min/1.73 m^2^ with structural or functional kidney abnormality) and AKI in the results for the cohort of the present study.

On the other hand, with regard to the relation between VTE and AKI, it should be interpreted carefully because the number of patients with VTE is small in the cohort for the present study; however, kidney failure in VTE has already been described in other publications and it might occur due to the concomitant heart failure (cardiorenal syndrome), hypoperfusion, and administration of contrast media for the diagnosis through angiotomography [32].

A recent study on the global overview of AKI (0 by 25 initiative) [33] showed that hypotension and shock were the most common causes of AKI in countries such as ours (medium-high per capita income stratum in 2014). Both entities have clinical conditions with multiple causes, including sepsis. In our hospital, hypotensive patients and/or septic shock patients are managed in the ICU; therefore, they were excluded from our study. Based on the third international consensus defining sepsis and septic shock published after our study design and data collection [34], the “infected patients” in our cohort who developed AKI have organ dysfunction and are, therefore, true septic patients according to the new consensus.

The present study contributes to bridging the gap in scientific research on the subject of AKI in Latin American and/or developing countries [15, 35, 36]. Addressing the epidemiology of AKI in nonsurgical patients is a great challenge, given the wide heterogeneity in study definition and design, whose results become difficult to compare; most original articles on AKI focus on cardiovascular surgery and critical care units [20, 36–39], whereas the others focus on specific clinical conditions, including pneumonia, renal transplantation, tropical diseases, and even the CA-AKI [40, 41].

Optimal patient inclusion and in-hospital prospective monitoring for EAKI incidence estimation and correct differentiation between patients with CKD without exacerbation on admission and patients with CA-AKI are among the strengths of this study. Although CA-AKI was not a study endpoint, remarkably, it is the leading cause of patient exclusion from our cohort and accounts for 19.8% (*n* = 239) of all patients evaluated. This issue will be the focus of further research.

Our study has several limitations. This study was single-center in design, which limits its external validity because disease behavior may vary depending on and according to healthcare center, region, or country [33]. Furthermore, no outcome monitoring was performed beyond the period of hospitalization. Therefore, key data, including the incidence of CKD after EAKI, mortality rate, and end-stage renal disease (ESRD), are unknown. Conversely, hydration status on admission was only assessed based on the attending physician's clinical criteria, and no objective evaluation was performed using tools such as bioelectrical impedance analysis.

Based on this study, we are currently working towards providing the patient wellness program with good practice guides for the prevention and detection of AKI, seeking to strengthen the “hospital free of preventable AKI” project. The AKI detection strategy used in our study can serve to guide other hospital centers in the promotion of pertinent medical interventions, such as how to maintain adequate hydration, improve the prescription writing habits of physicians (avoiding the indiscriminate use of nephrotoxic drugs), and solicit the evaluation of a nephrology specialist in a timely manner.

In conclusion, the incidence of AKI detected within five days of hospitalization in the nonsurgical population and without apparent AKI on admission is similar to that reported in the international literature and is associated with prolonged hospital stay, in-hospital death, and dialysis requirement. The associated risk factors include CKD at advanced stages (NFK-KDOQI stages 3, 4, and 5), prehospital treatment with nephrotoxic drugs, and VTE. Research on this topic must be encouraged to strengthen the epidemiological data and, in turn, to generate individualized strategies in each region to avoid preventable deaths from AKI.

## Supplementary Material

Supplementary Table 1 shows the operational definitions used in our study for prehospital and intrahospital nephrotoxic drugs.
Supplementary Table 2 shows the median of the hospital stay according to the condition at discharge (alive or dead) and if EAKI was developed.

## Figures and Tables

**Table 1:** Population characteristics.

Variable	Total	Early acute kidney injury		*p* value
*n* (%) or median (IQR)	*n* = 400	No (*n* = 336)	Yes (*n* = 64)	
Age (years)	65 (49–77)	64 (47–77)	68 (58–81)	0.033
Sex (male)	180 (45)	151 (44.9)	29 (45.3)	0.776
Weight (kg)	64.4 (54.9–75)	64.1 (55–75)	66 (54.4–74.1)	0.993
Anemia	138 (34.5)	111 (33.0)	27 (42.19)	0.158
History of diabetes mellitus	91 (22.7)	69 (20.5)	22 (34.4)	0.015
History of AHT	194 (48.5)	155 (46.1)	39 (60.9)	0.030
History of cirrhosis	10 (2.5)	8 (2.4)	2 (3.13)	0.727
History of heart failure	71 (17.7)	57 (16.9)	14 (21.8)	0.346
History of coronary heart disease	44 (11)	34 (10.1)	10 (15.6)	0.197
History of rheumatologic disease	40 (10)	36 (10.7)	4 (6.3)	0.275
CKD^*∗*^ at admission	243 (60.7)	149 (44.3)	19 (29.7)	<0.001
Stage 1 (>90 mL/min/1.73 m^2^)	6 (1.5)	6 (1.5)	0 (0)	
Stage 2 (60–90 mL/min/1.73 m^2^)	162 (40.5)	143 (42.6)	19 (29.7)	
Stage 3 (30–59 mL/min/1.73 m^2^)	65 (16.2)	46 (13.7)	19 (29.7)	
Stage 4 (15–29 mL/min/1.73 m^2^)	6 (1.5)	1 (0.3)	5 (7.8)	
Stage 5 (<15 mL/min/1.73 m^2^)	4 (1)	2 (0.6)	2 (13.1)	
In-hospital treatment with nephrotoxic drugs	**376 (94)**	**315 (93.7)**	**61 (95.3)**	**0.630**
Contrast	81 (20.2)	68 (20.2)	13 (20.3)	
NSAIDs	44 (11)	40 (11.9)	4 (6.3)	
Vancomycin	17 (4.2)	14 (4.2)	3 (4.7)	
Proton pump inhibitor	314 (78.5)	264 (78.6)	50 (78.1)	
Quinolones	2 (0.5)	2 (0.6)	0 (0)	
Aminoglycosides	1 (0.25)	0 (0)	1 (1.6)	
Polymyxin B	1 (0.25)	1 (0.3)	0 (0)	
IECA/ARAII	168 (42)	139 (41.4)	29 (45.3)	
Furosemide	125(31.2)	95 (28.3)	30 (46.9)	
Potassium-sparing diuretics	32 (8)	26 (7.7)	6 (9.4)	
Thiazide diuretics	12 (3)	11 (3.3)	1 (1.6)	
Prehospital treatment with potentially nephrotoxic drugs	**239 (59.7)**	**190 (56.5)**	**49 (76.6)**	**0.003**
Statins	70 (17.5)	61 (18.2)	9 (14.6)	
NSAIDs	54 (13.5)	44 (13.1)	10 (15.6)	
Quinolones	1 (0.25)	1 (0.3)	0 (0)	
Aminoglycosides	1 (0.25)	0 (0)	1 (1.6)	
IECA/ARAII	172 (43)	140 (41.7)	32 (50.0)	
Furosemide	84 (21)	62 (18.5)	22 (34.4)	
Potassium-sparing diuretics	33 (8.25)	28 (8.3)	5 (7.8)	
Thiazide diuretics	33 (8.25)	25 (7.4)	8 (12.5)	
Primary diagnosis on admission				**<0.001**
Bacterial infection	172 (43)	149 (44.4)	23 (35.9)	
Cardiovascular disease	75 (18.7)	58 (17.3)	17 (26.6)	
Chronic pulmonary and pleural disease	59 (14.7)	53 (15.8)	6 (9.4)	
Endocrine disease	18 (4.5)	12 (3.6)	6 (9.4)	
Venous thromboembolism	19 (4.7)	13 (3.9)	6 (9.4)	
Others^†^	57 (14.2)	51 (15.2)	6 (9.4)	
Hydrated	119 (29.7)	104 (30.9)	15 (23.4)	0.228
Sepsis^‡^	159 (39.7)	137 (40.8)	22 (34.4)	0.338
Nephrotic syndrome	4 (1)	2 (0.6)	2 (3.1)	0.062
Isolated proteinuria	51 (12.7)	40 (11.9)	11 (17.2)	0.246
Days of hospital stay	7 (4–11)	6 (4–10)	8 (5–14)	0.009
ICU requirement	34 (8.5)	25 (7.4)	9 (14.1)	0.082
Renal replacement therapy	4 (1)	1 (0.3)	3 (4.7)	<0.001
Condition on discharge (death)	30 (7.5)	16 (4.8)	14 (21.9)	<0.001

^*∗*^Calculated using the chronic kidney disease epidemiology collaboration (CKD-EPI) equation and classified based on NFK-KDOQI guideline.

^†^Including gastrointestinal disease, rheumatologic disease, glomerular and tubulointerstitial disease, nonneoplastic hematologic disease, solid tumors and hematological malignancies, and viral infections.

^‡^Defined as systemic inflammatory response syndrome (SIRS) with septic focus.

**Table 2:** Risk factors associated with EAKI, bivariate analysis.

Variable	OR	95% CI	*p* value
CKD^*∗*^ stages 1 and 2	0.93	[0.44–1.93]	0.824
CKD^*∗*^ stages 3, 4, and 5	4.00	[2.13–7.45]	<0.001
Prehospital treatment with nephrotoxic drugs	2.63	[1.38–5.25]	0.002
In-hospital treatment with nephrotoxic drugs	1.35	[0.39–7.31]	0.630
Contrast media	1.00	[0.47–2.00]	0.990
History of DM	2.06	[1.09–3.80]	0.013
History of AHT	1.82	[1.02–3.28]	0.030
History of cirrhosis	1.32	[0.13–6.84]	0.730
History of heart failure	1.37	[0.65–2.73]	0.350
History of rheumatologic disease	0.55	[0.14–1.64]	0.260
History of coronary heart disease	1.64	[0.68–3.65]	0.197
Nephrotic syndrome	5.39	[0.38–75.1]	0.062
Age	1.02	[1.00–1.03]	0.019
Hydration status	0.68	[0.34–1.30]	0.230
Sepsis	0.38	[0.76–1.37]	0.338
Venous thromboembolism at admission	2.57	[0.77–7.59]	0.057
Cardiovascular disease at admission	1.73	[0.87–3.33]	0.086
Chronic pulmonary and pleural disease at admission	0.55	[0.18–1.37]	0.185

^*∗*^Calculated using the chronic kidney disease epidemiology collaboration (CKD-EPI) equation and classified based on NFK-KDOQI guidelines.

**Table 3:** Risk factors associated with EAKI, multivariate analysis.

Variable	OR	95% CI	*p* value
CKD^*∗*^ stages 1 and 2	0.83	[0.39–1.75]	0.628
CKD^*∗*^ stages 3, 4 and 5	3.56	[1.55–8.18]	0.003
Prehospital treatment with nephrotoxic drugs	2.21	[1.12–4.36]	0.022
Venous thromboembolism at admission	5.05	[1.59–16.0]	0.006
Cardiovascular disease at admission	1.23	[0.58–2.63]	0.592
Hydration status	0.55	[0.27–1.10]	0.077
Age	1.00	[0.98–1.02]	0.627
Sepsis	1.12	[0.56–2.23]	0.752

^*∗*^Calculated using the chronic kidney disease epidemiology collaboration (CKD-EPI) equation and classified based on NKF-KDOQI guidelines.
