# Supplementary material for: Incidence and Risk Factors for Early Acute Kidney Injury in Nonsurgical Patients: A Cohort Study
Source: Int J Nephrol. 2017 Apr 11;2017:5241482. doi: 10.1155/2017/5241482 (PMC5405385; doi:10.1155/2017/5241482)
Supplement: Supplementary file 1 — Supplementary Table 1 shows the operational definitions used in our study for prehospital and intrahospital nephrotoxic drugs. Supplementary Table 2 shows the median of the hospital stay according to the condition at discharge (alive or dead) and if EAKI was developed. [file 5241482.f1.zip › Supplement Table 1_IJN_1894695.docx]

**Table 1. Operational definitions for prehospital and intra-hospital nephrotoxic drugs**

| **Intra-hospital nephrotoxic drugs** | |
| --- | --- |
| Iodine contrast media | Administration of contrast media to carry out any procedure within the first 48 hours of hospitalization. |
| NSAIDs | Administration of at least 1 daily intra-hospital dose within the first 48 hours, it is not valid if it was being received during the last 7 days prior to hospitalization. |
| Vancomycin |  |
| Proton pump inhibitor |  |
| Fluoroquinolone |  |
| Aminoglycoside |  |
| Polymyxin B |  |
| I-ECA or ARA -II | Administration of at least 1 daily intra-hospital dose (oral or IV) within the first 48 hours, being unimportant if it was being received at home prior to hospitalization. |
| Potassium-sparing diuretic |  |
| Thiazide diuretic |  |
| Furosemide |  |

| **Prehospital nephrotoxic drugs** | |
| --- | --- |
| Statin | Daily consumption of high power statin within the last three months prior to hospitalization (rosuvastatin ≥10 mg/day or atorvastatin ≥20 mg/day or simvastatin ≥ 40 mg/day ) |
| NSAIDs | Consumption of at least 1 daily dose within the last 7 days prior to hospitalization |
| Fluoroquinolone |  |
| Aminoglycoside |  |
| I-ECA or ARA -II | Consumption of at least 1 daily dose within the last 3 months prior to hospitalization |
| Furosemide |  |
| Potassium-sparing diuretic |  |
| Thiazide diuretic |  |
