# Supplementary material for: Incidence and Risk Factors for Early Acute Kidney Injury in Nonsurgical Patients: A Cohort Study
Source: Int J Nephrol. 2017 Apr 11;2017:5241482. doi: 10.1155/2017/5241482 (PMC5405385; doi:10.1155/2017/5241482)
Supplement: Supplementary file 1 — Supplementary Table 1 shows the operational definitions used in our study for prehospital and intrahospital nephrotoxic drugs. Supplementary Table 2 shows the median of the hospital stay according to the condition at discharge (alive or dead) and if EAKI was developed. [file 5241482.f1.zip › Supplement Table 2_IJN_1894696.docx]

**Table 2 Length of hospital stay by E-AKI* and condition on discharge**

| **Variable (E-AKI and condition on discharge)** | **Total**  **n=400** | **Length of stay**  **Median (IQR)** |
| --- | --- | --- |
| E-AKI **= No**  Condition on discharge **= Alive** | n=320 | 6(4-10) |
| E-AKI **= No**  Condition on discharge **= Death** | n=16 | 8.5 (6-11) |
| E-AKI **= Yes**  Condition on discharge **= Alive** | n=50 | 8 (5-14) |
| E-AKI **= Yes**  Condition on discharge **= Death** | n=14 | 8 (3-17) |

*Early acute kidney injury
